# Pharmacological Inhibition of CXCR2 Chemokine Receptors Modulates Paraquat-Induced Intoxication in Rats

**DOI:** 10.1371/journal.pone.0105740

**Published:** 2014-08-25

**Authors:** Kesiane M. Costa, Izaque S. Maciel, Luiza W. Kist, Maria M. Campos, Maurício R. Bogo

**Affiliations:** 1 Postgraduate Program in Medicine and Health Sciences, Pontifical Catholic University of Rio Grande do Sul, Porto Alegre/RS, Brazil; 2 Laboratory of Genomics and Molecular Biology, Faculty of Biosciences, Pontifical Catholic University of Rio Grande do Sul, Porto Alegre/RS, Brazil; 3 Institute of Toxicology and Pharmacology, Pontifical Catholic University of Rio Grande do Sul, Porto Alegre/RS, Brazil; 4 Faculty of Dentistry, Pontifical Catholic University of Rio Grande do Sul, Porto Alegre/RS, Brazil,; 5 Postgraduate Program in Cellular and Molecular Biology, Pontifical Catholic University of Rio Grande do Sul, Porto Alegre/RS, Brazil; University of Florida, United States of America

## Abstract

Paraquat (PQ) is an agrochemical agent commonly used worldwide, which is allied to potential risks of intoxication. This herbicide induces the formation of reactive oxygen species (ROS) that ends up compromising various organs, particularly the lungs and the brain. This study evaluated the deleterious effects of paraquat on the central nervous system (CNS) and peripherally, with special attempts to assess the putative protective effects of the selective CXCR2 receptor antagonist SB225002 on these parameters. PQ-toxicity was induced in male Wistar rats, in a total dose of 50 mg/kg, and control animals received saline solution at the same schedule of administration. Separate groups of animals were treated with the selective CXCR2 antagonist SB225002 (1 or 3 mg/kg), administered 30 min before each paraquat injection. The major changes found in paraquat-treated animals were: decreased body weight and hypothermia, nociception behavior, impairment of locomotor and gait capabilities, enhanced *TNF-α* and *IL-1β* expression in the striatum, and cell migration to the lungs and blood. Some of these parameters were reversed when the antagonist SB225002 was administered, including recovery of physiological parameters, decreased nociception, improvement of gait abnormalities, modulation of striatal *TNF-α* and *IL-1β* expression, and decrease of neutrophil migration to the lungs and blood. Taken together, our results demonstrate that damage to the central and peripheral systems elicited by paraquat can be prevented by the pharmacological inhibition of CXCR2 chemokine receptors. The experimental evidence presented herein extends the comprehension on the toxicodynamic aspects of paraquat, and opens new avenues to treat intoxication induced by this herbicide.

## Introduction

Paraquat (PQ) dichloride is a fast-acting, non-selective bipyridylium herbicide, widely used in many countries worldwide due to the low costs and effectiveness against a range of weeds. The accidental or intentional poisoning with this agent has been associated with fatal cases since there is a lack of effective treatments to intoxication (for review see: [1]). In fact, the mechanisms implicated in the toxicity caused by paraquat exposure are not yet completely understood [2]. Regardless, it has been demonstrated that paraquat effects are primarily allied with oxidative stress through the production of reactive oxygen species (ROS) [3],[4]. After exposure, the herbicide can be detected in several organs including heart, kidneys, liver, thymus, and lungs [5]. The concentration in lungs is commonly highly elevated, as a consequence of its affinity for type 2 pneumocytes. Of note, acute lung injury with fibrosis and massive cell infiltration in the alveolar spaces can be observed after intoxication with a single dose of paraquat [6].

Chemokines are a subset of cytokines that selectively regulate the recruitment of leukocytes to inflammatory sites, mainly through the activation of G-protein-coupled receptors [7],[8]. These receptors are classified into different families according to the type of chemokines they bind (CC, CXC, C, or CX_3_C) (for review see: [9]). CXC chemokines are especially involved in migration of polymorphonuclear leukocytes (PMNs), and the prototype of this group is interleukin-8 (IL-8/CXCL8), which binds to the CXCR1/2 receptors [10]. Of note, it has been demonstrated that paraquat is able to activate a set of genes related to immune response (IL-10, CXL10, and CXL11), according to evaluation of the human keratinocyte cell line HaCaT [11].

In 1998, White et al. [12] described the first non-peptide, selective, and competitive CXCR2 receptor antagonist, named SB225002, which was found effective in preventing a series of IL-8-mediated inflammatory responses, both *in vitro* and *in vivo*. By using an *in vivo* model of mouse colitis induced by 2,4,6-trinitrobenzene sulfonic acid (TNBS), Bento et al. [13] demonstrated that intraperitoneal (i.p.) administration of SB225002, in doses as low as 1 mg/kg, was able to prevent several inflammatory parameters, such as neutrophil migration to the colon tissue. Furthermore, i.p. treatment with SB225002 resulted in a marked and long-lasting inhibition of acute or chronic nociception in mice [14]. More recently, SB225002 was demonstrated to be effective in preventing the functional, inflammatory, and nociceptive alterations related to cyclophosphamide-induced cystitis in rats when dosed i.p., alone or in combination with the transient receptor potential vanilloid 1 (TRPV1) antagonist SB266791 [15]. Another recent study demonstrated that spinal injection of SB225002 (5 and 20 µg/site) significantly inhibited the mechanical allodynia caused by CXL1/KC in mice [8].

The present study examined, for the first time, the possible beneficial effects of SB225002 in a rat model of paraquat intoxication, induced by repeated exposure to low doses of this herbicide. Interestingly, our results revealed that pharmacological inhibition of CXCR2 receptors was allied to improvement of both central and peripheral complications induced by paraquat, thus contributing to clarify its mechanisms of toxicity.

## Materials and Methods

### Ethics Statement

Experiments were conducted in accordance with current guidelines for the care of laboratory animals and ethical guidelines for the investigation of experimental pain in conscious animals [16]. All the experimental procedures were approved by the Animal Ethics Committee of Pontifícia Universidade Católica do Rio Grande do Sul (RS) (Protocol Number: CEUA 12/00295).

### Animals

Male Wistar rats (weighing 180 g at the beginning of experiments) used in this study were obtained from the Central Animal House of Universidade Federal de Pelotas (UFPEL, Brazil). The animals were housed in groups of four and maintained in controlled temperature (22±1°C) and humidity (60–70%), under a 12 h light-dark cycle (lights on 07:00 AM). Food and water were available *ad libitum*. Animals were acclimatized to the laboratory for at least 1 h before testing and all the tests were performed between 9:00 AM and 5:00 PM. The number of animals and intensity of noxious stimuli used were the minimum necessary to provide consistent effects of the treatments. Analgesics were not administered to avoid any possible interference with the obtained results. In all experimental protocols, the animals were monitored twice daily for signals of severe distress. The criteria adopted to decide on euthanasia included the occurrence of tremors, convulsions, and/or chromodacryorrhea. The euthanasia was performed by deep anesthesia with isoflurane.

### General protocols of treatment

**Figure 1:**
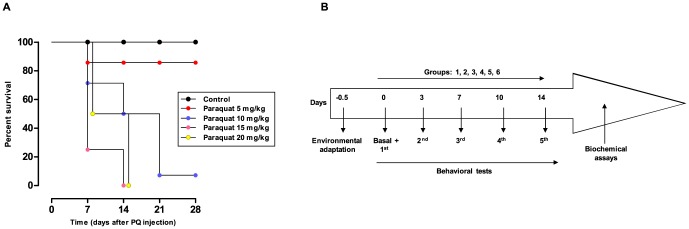
Paraquat survival curve and general schedule of treatment used in the study. (A) Survival curve for cumulative paraquat administration through 28 days, given at different doses [5 (n = 4), 10 (n = 14), 15 (n = 4), and 20 (n = 6) mg/kg], twice a week. (B) The animals received five i.p. applications (at 0, 3, 7, 10, and 14 days) of vehicle/saline (10 ml/kg; group 1), SB225002 (1 and 3 mg/kg; groups 2 and 3)/saline, vehicle/paraquat (10 mg/kg; group 4), or SB225002 (1 and 3 mg/kg)/paraquat (10 mg/kg); groups 5 and 6, respectively. Behavioral tests were performed at days 0, 7, and 14, which corresponded to the basal timepoint, and the 3^rd^ or the 5^th^ application of drugs. In some cases, the behavioral measurements were also carried out under basal conditions (prior to any treatment).

Initially, a total of 28 rats received i.p. applications of paraquat at different doses [5 (n = 4), 10 (n = 14), 15 (n = 4), and 20 (n = 6) mg/kg] [17], twice a week for 28 days, in order to determine the appropriate dose for the next experiments. A separate group of animals received saline solution (n = 8) as control (Figure 1A). The number of animals used to determine the experimental design was 36 rats. Next, on the basis of the previous set of experiments and based on the literature [18], animals were provided with paraquat (10 mg/kg), twice a week for 14 days, at a total dose of 50 mg/kg. The animals used in the behavioral analysis were further used in molecular and biochemical analysis. Briefly, rats were randomly divided into six groups comprising: (A) vehicle/saline (1% Tween-80 in 0.9% NaCl solution; 10 ml/kg), with a total number of 15 animals; (B) SB225002 (1 mg/kg)/saline, with a total number of six animals; (C) SB225002 (3 mg/kg)/saline, with a total number of four animals; (D) vehicle/paraquat, with a total number of 20 animals; (E) SB225002 (1 mg/kg)/paraquat, with a total number of nine animals and (F) SB225002 (3 mg/kg)/paraquat, with a total number of 13 animals. The number of animals used in the behavioral, molecular, and biochemical analysis was 67 rats. Therefore, the total number of animals used in this study was 103 rats. The doses of SB225002 were selected on the basis of previous publications [14]. The antagonist (or the vehicle) was administered 30 min before each paraquat or saline application. The protocol of the treatments is outlined in Figure 1B.

### Body weight measurement

Rat body weight was recorded (in g) using a digital balance. All the animals were weighed once a day at the basal timepoint, and before the 2^nd^, 3^rd^, 4^th^, and 5^th^ applications. The values are expressed as the difference between the initial weights minus the weight values on the day of measurement.

### Body temperature assessment

The rectal temperature was recorded (in °C) using a commercially available thermometer. After recording the initial rectal temperature (basal measurement; in °C), the body temperature was evaluated on days corresponding to the 2^nd^, 3^rd^, 4^th^, and 5^th^ applications at two different timepoints: immediately before, and 1 h after the treatments. The values are expressed as the difference between the temperatures before and after application.

### Nociceptive responses

The method adopted in the present study was similar to that described previously [19],[20],[21],[22], with minor modifications. The animals were analyzed in their cages in order to minimize the potential variations in the behavioral responses. After the treatments, the animals were assessed in two different approaches: (i) the spontaneous behavior was measured 25 min after treatment for 5 min; (ii) the spontaneous behavior was measured after 55 min for 5 min, over a total period of a 1 h. The behavioral alterations were scored according to the following scale: 0  =  absence of any signal, with normal eyes; 1 – piloerection; 2 – stretching; 1 – hump-backed position; 1 – half-closed eyes; 2 – completely closed eyes. All the experiments were performed in a blinded manner, each experimenter scoring four rats in parallel. The scores were registered over each 5-min period, and summed for an overall pain score (maximum score =  6).

### Open-field test

To analyze the locomotor activity, the animals were evaluated in the open-field test, according to the methodology described before [23], with minor modifications. The experiments were conducted in a sound-attenuated room, under low-intensity light. Rats were individually placed in the center of a plywood box (34.5 cm height×249 cm circumference×77.5 cm radius), with the floor divided into 17 squares. Duration of grooming (s) was cumulatively registered over 5 min. The number of rearing behaviors and fecal boluses was also recorded. Additionally, the number of squares crossed with the four paws was registered. All the parameters were measured at the basal timepoint and after the 3^rd^ and 5^th^ applications.

### Beam-walking test

To assess the locomotor skills, the animals were evaluated in the beam-walking test. The methodology used was the same described previously [24]. Each rat was positioned in the center of a wooden narrow bridge (3.5 cm wide and 85 cm long), suspended between two platforms at an angle of 8° [25]. The time taken to cross the beam (in seconds, s), and the number of paw slips and falls was registered during 3 min. Each animal was acclimatized on the platform on three consecutive days before the experiments. The measurements were made at the basal timepoint and after the 3^rd^ and the 5^th^ applications.

### Footprint test

To determine the gait abnormalities induced by paraquat, the animals were evaluated in the footprint test according to the methodology described by Richter et al. [26] with minor modifications, at the baseline and after the 3^rd^ and the 5^th^ applications of the herbicide. The rats were allowed to walk through an 85-cm-long, 5-cm-wide runway corridor, with a darkened cage at the end of the corridor. The floor of this corridor was covered with a new white absorbing paper for each run. The animals were first trained to pass straight forward through the corridor on three consecutive days before the experiments. After the training, the paws were colored with different colors (red for the forepaws and black for the hindpaws), and the rats were placed into the corridor. For footprint analysis, both the initial and the final 10 cm of the stamped paper was excluded to avoid false-positive results [27]. Two distinct parameters were considered for analysis: (i) the stride length was measured as the average distance between the extremity of third toes of the forepaws and hindpaws, for both left and right paws; (ii) the step frequency was evaluated according to the total number of footprints. The averages obtained by two blinded researchers were considered as a duplicate.

### Gene expression analysis by quantitative real-time RT-PCR (RT-qPCR)

Following the behavioral tests assessments, the animals were euthanized by deep inhalation of isoflurane and all the right and left striatum was collected. Total RNA was isolated, quantified by spectrophotometry (A260/280 nm) and after treated with deoxyribonuclease I to eliminate genomic DNA contamination. The cDNA was synthesized from 1 µg total RNA. Quantitative PCR was performed using SYBR Green to detect double-stranded cDNA synthesis. Reactions were done in a volume of 25 µL using 12.5 µL of diluted cDNA, containing a final concentration of 0.2x SYBR Green, 100 µM dNTP, 1x PCR Buffer, 3 mM MgCl_2_, 0.25 U Taq DNA Polymerase, 0.5 M betaine, and 200 nM of *COX-2* [28], *IL-1β*, *TNF-α* [29], *NF-κB1* [30], or *Rpl13α* [31] primers (Table 1). The PCR cycling conditions were: an initial polymerase activation step for 5 min at 95°C, 40 cycles of 15 s at 95°C for denaturation, 35 s at 60°C for annealing, and 15 s at 72°C for elongation. At the end of the cycling protocol, a melting-curve analysis was included and fluorescence measured from 60 to 99°C and showed in all cases one single peak. *Rpl13α* was used as an endogenous control. Relative expression levels were determined with 7500 and 7500 Fast Real-Time PCR Systems Software v.2.0.6 (Applied Biosystems). The efficiency per sample was calculated using LinRegPCR 2012.3 Software (http://LinRegPCR.nl). Relative mRNA expression levels were determined using the 2^-ΔΔCT^ method [32].

**Table 1:** Primer sequences for RT-qPCR experiments.

Gene	Forward primer	Reverse primer
*COX-2* ^a^	5′-CCCCAAGGCACAAATATGATG-3′	5′-CCTCGCTTCTGATCTGTCTTGA-3′
*IL-1β* ^b^	5′-GGACAGAACATAAGCCAACAA-3′	5′-CTTTCATCACACAGGACAGGT-3′
*NF-κB1* ^c^	5′-CAGCTCTTCTCAAAGCAGCA-3′	5′-TCCAGGTCATAGAGAGGCTCA-3′
*Rpl13α* ^d^	5′-ACAAGAAAAAGCGGATGGTG-3′	5′-TTCCGGTAATGGATCTTTGC-3′
*TNFα* ^b^	5′-AGCAGATGGGCTGTACCTTAT-3′	5′-GCTGACTTTCTCCTGGTATGA -3′

According to ^a^Hanafy et al., 2012; ^b^Singh et al., 2012; ^c^Kireev et al., 2012; ^d^Bonefeld et al., 2008.

### Hematologic parameters

After the behavioral assessments, the animals were euthanized by deep inhalation of isoflurane and the blood was collected. Immediately after, a small drop of blood was taken for smear evaluation using May-Grunewald-Giemsa staining [33]. Differential counts (neutrophils, eosinophils, basophils, lymphocytes, monocytes, and immature cells) were estimated under a ×40 objective, by counting 100 cells. Representative pictures were captured.

### Myeloperoxidase (MPO) activity

Neutrophil recruitment to the rat lung was measured by means of tissue MPO activity, according to the method described by Souza et al. [34], with minor modifications. After euthanasia by deep inhalation of isoflurane, the lungs of animals were removed and immediately stored at −80°C. The tissues were homogenized in 5% (w/v) EDTA/NaCl buffer (pH 4.7) and centrifuged at 4000 rpm for 25 min, at 4°C. The pellet was resuspended in 0.5% hexadecyltrimethyl ammonium bromide buffer (pH 5.4), and the samples were re-centrifuged (4000 rpm, 25 min, 4°C). Twenty-five microliters of the supernatant were used for the MPO assay. The enzymatic reaction was assessed with 1.6 mM tetramethylbenzidine, 80 mM NaPO_4_, and 0.3 mM hydrogen peroxide. The absorbance was measured at 595 nm, and the results are expressed in optical density (OD) per milligram of tissue.

### Statistical analysis

Results are presented as mean ± standard error mean (SEM). The statistical comparison of the data was performed by one-way analysis of variance (ANOVA), followed by Bonferroni's post-hoc test. Comparison of the survival curves was performed using the Log-rank test. *P* values less than 0.05 (p<0.05) were considered as indicative of significance.

### Drugs and Reagents

The following drugs and reagents were used: paraquat was purchased from Syngenta Limited. N-(2-hydroxy-4-nitrophenyl)-N9-(2-bromophenyl) Urea (SB225002) was synthesized as described before [12] and provided by Universidade Federal de Santa Catarina (UFSC). SB225002 was identified by comparing the 1H NMR data with those published beforehand (Yield 70%, m.p.  =  193–194 _C. 1H NMR (400 MHz) (ppm) (DMSO-d6) 11.05 (br. s, 1H), 9.48 (s, 1H), 9.12 (s, 1H), 8.36 (d, 1H), 7.93 (d, 1H), 7.75 (dd, 1H), 7.69 (s, 1H), 7.63 (d, 1H), 7.36 (t, 1H), 7.03 (t, 1H); IR (cm_1) 3364, 3212, 1692, 1588, 1536, 1507, 1430, 1306, 1267, 1212, 1086, 746, 649. LC–MS-ESI (m/z) 350 (M-H)-. SB225002 was dissolved in 1% Tween-80 in 0.9% NaCl solution.

## Results

### Dose-related toxicity of paraquat and experimental design

This experimental set was designed to characterize the behavioral changes and mortality after treatment with different doses of paraquat in order to define the ideal scheme of treatment for the next experiments. Thus, different doses of paraquat [5 (n = 4), 10 (n = 14), 15 (n = 4), and 20 (n = 6) mg/kg] were administered to rats, twice a week over 28 days and the percentage of survival (Figure 1A) was registered.

The animals treated with 5 mg/kg per dose of paraquat did not show significant changes in behavioral tests, displaying low toxicity, as was observed in the negative control group (treated with saline). In the 10 mg/kg paraquat group, significant time-related changes were observed in the behavioral tests, and 7/14 of animals died within the first 14 days. With paraquat doses of 15 mg/kg and 20 mg/kg, all the animals died within the first 14 days of treatment. Therefore, further experiments were performed using the dose of 10 mg/kg (sub-lethal dose), twice a week over 14 days, with a total cumulative dose of 50 mg/kg (Figure 1B).

### Physiological changes evoked by paraquat: effects of SB225002

**Figure 2:**
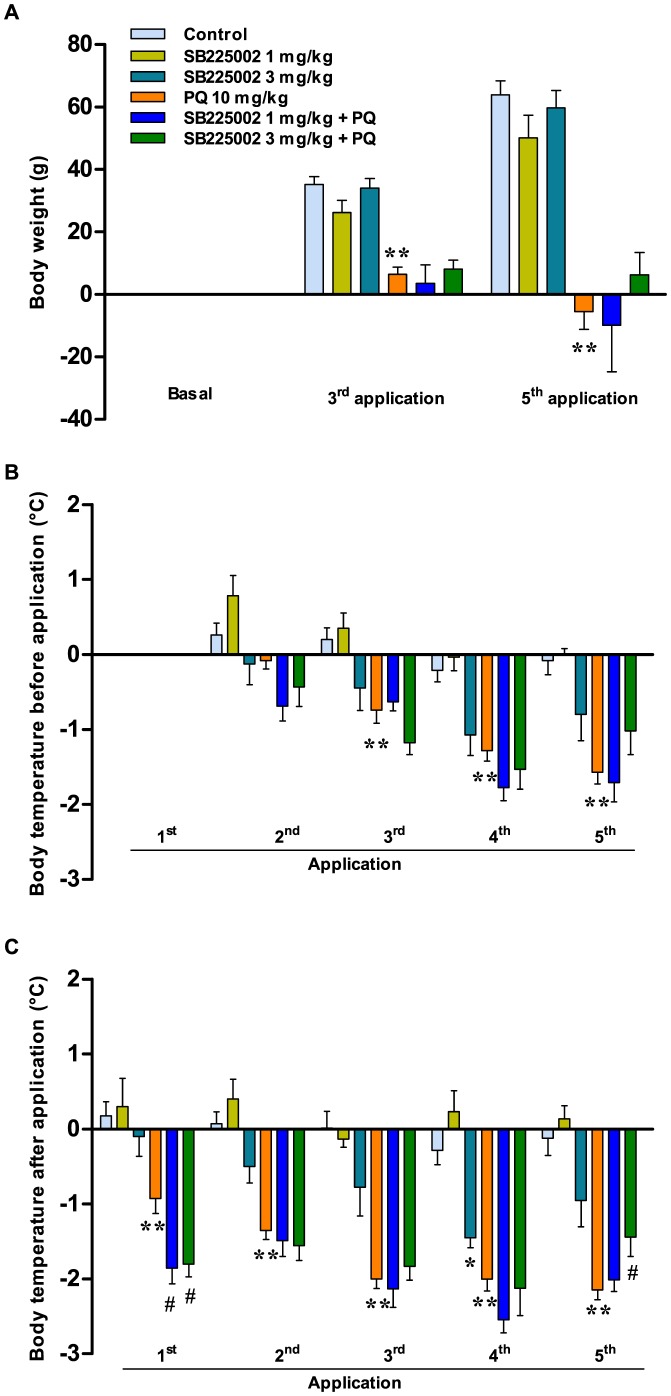
Physiological changes elicited by paraquat: effects of SB225002. (A) Changes in body weight (Δ grams), before the 3^rd^ and 5^th^ application; (B) variation of body temperature (Δ °C), measured immediately before treatments; (C) variation of body temperature measured 1 h after each application. Each column represents the mean ± SEM of 4–18 animals per group. ^*^p<0.05 and ^**^p<0.01 significantly different from control (vehicle/saline) group; ^#^p<0.05 significantly different from vehicle/paraquat group (PQ) (ANOVA followed by Bonferroni's post-hoc test).

The treatment with 10 mg/kg per dose of paraquat, with a total dose of 50 mg/kg within 14 days, resulted in a significant and time-related reduction of body weight (p<0.01) in comparison to the control vehicle/saline group. This effect was partially, although not significantly, prevented by pre-treating animals with the selective CXCR2 antagonist SB225002 (3 mg/kg), as observed at the 5^th^ application (Figure 2A).

The rectal temperature was checked at two distinct timepoints, immediately before and 1 h after treatments. The administration of paraquat resulted in a time-dependent and significant reduction of body temperature in both cases (p<0.01). This effect was significant from the 3^rd^ application, when the temperature was checked before each administration of paraquat (Figure 2B), and at all the experimental times following paraquat dosage (Figure 2C). Of note, the treatment with SB225002 (3 mg/kg) was able to significantly reverse the body temperature decrease, according to assessment after the last application of paraquat (p<0.05; Figure 2C). SB225002 (either 1 or 3 mg/kg) did not elicit any significant effect (Figures 2A and 2B), except by a significant decrease of rectal temperature after 1 h of antagonist application (4^th^ application, 3 mg/kg SB225002), an action that was reversed in the subsequent measurement (5^th^ application, 3 mg/kg SB225002) (Figure 2C).

### Effects of treatment with SB225002 on paraquat-elicited nociception

**Figure 3:**
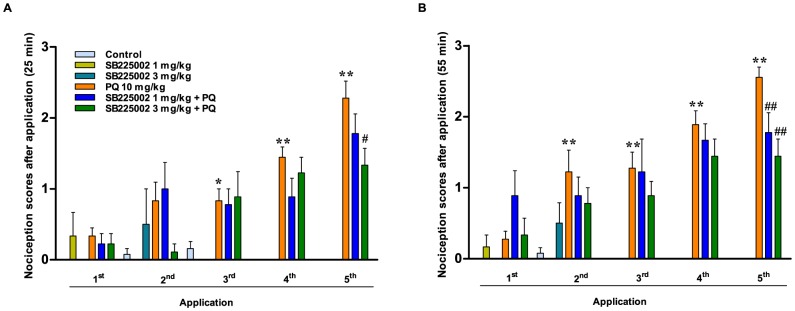
Behavioral scores of nociception. Assessment of behavioral scores at 25 min (A) or 55 min (B) after each treatment. Data is plotted as the cumulative scores of nociception over 5 min. Each column represents the mean ± SEM of 4–18 animals per group. ^*^p<0.05 and ^**^p<0.01 significantly different from control (vehicle/saline) group; ^#^p<0.05 and ^##^p<0.01 significantly different from vehicle/paraquat group (PQ) (ANOVA followed by Bonferroni's post-hoc test).

We also investigated whether nociceptive changes induced by paraquat might be prevented by SB225002. Intoxication with paraquat was associated with time-dependent nociceptive behavior, which was significantly different from vehicle/saline control animals, as recorded at 25 (Figure 3A) or 55 min (Figure 3B) following the herbicide application. In either protocol, the maximal effects of paraquat was observed at the 5^th^ application (p<0.01). Of note, the administration of SB225002 (3 mg/kg) was able to significantly reduce paraquat-evoked nociception, at either 25 (p<0.05) or 55 min (p<0.01) timepoints (Figure 3A and 3B). However, 1 mg/kg SB225002 displayed a significant effect on paraquat-induced nociception only at 55 min of evaluation (p<0.01).

### Effects of paraquat administration in the open-field paradigm

**Figure 4:**
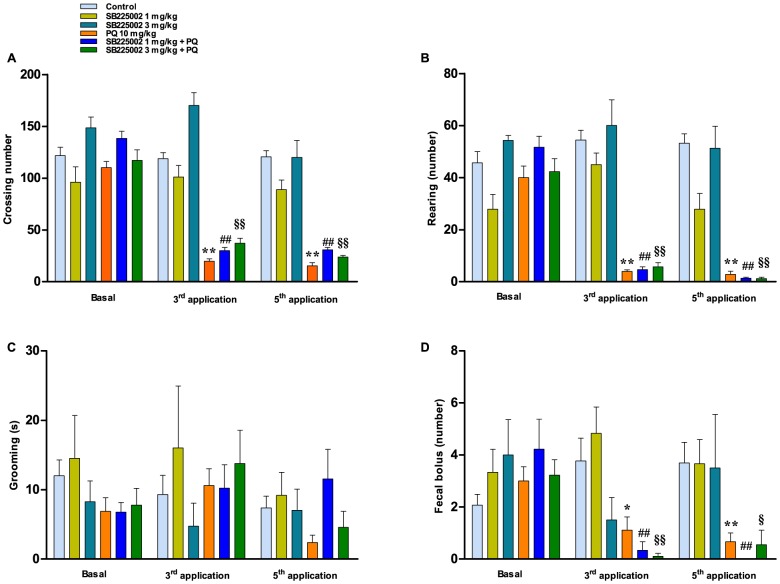
Evaluation of paraquat effects in the open-field test. (A) Number of crossed squares; (B) number of rearings; (C) facial grooming in s; (D) number of fecal boluses. Each column represents the mean ± SEM of 4–18 animals per group. ^*,§^p<0.05; ^**,##,§§^p<0.01 compared with the basal measurements of the same experimental group (ANOVA followed by Bonferroni's post-hoc test).

In these experiments, we analyzed to what extent the treatment with paraquat might affect locomotor and exploration tasks in the open-field apparatus. Paraquat-intoxicated animals displayed significant reductions of crossing (Figure 4A) and rearing counts (Figure 4B) at both the 3^rd^ and 5^th^ applications (p<0.01). Although grooming behavior was not significantly affected (Figure 4C; p>0.05), paraquat administration caused a significant decrease in the number of fecal boluses (Figure 4D; p<0.01). SB225002 (1 or 3 mg/kg) did not display any unspecific effect, and this antagonist failed to significantly modify the effects of paraquat in the open-field model (Figure 4A–4D; p>0.05).

### Assessment of motor coordination

**Figure 5:**
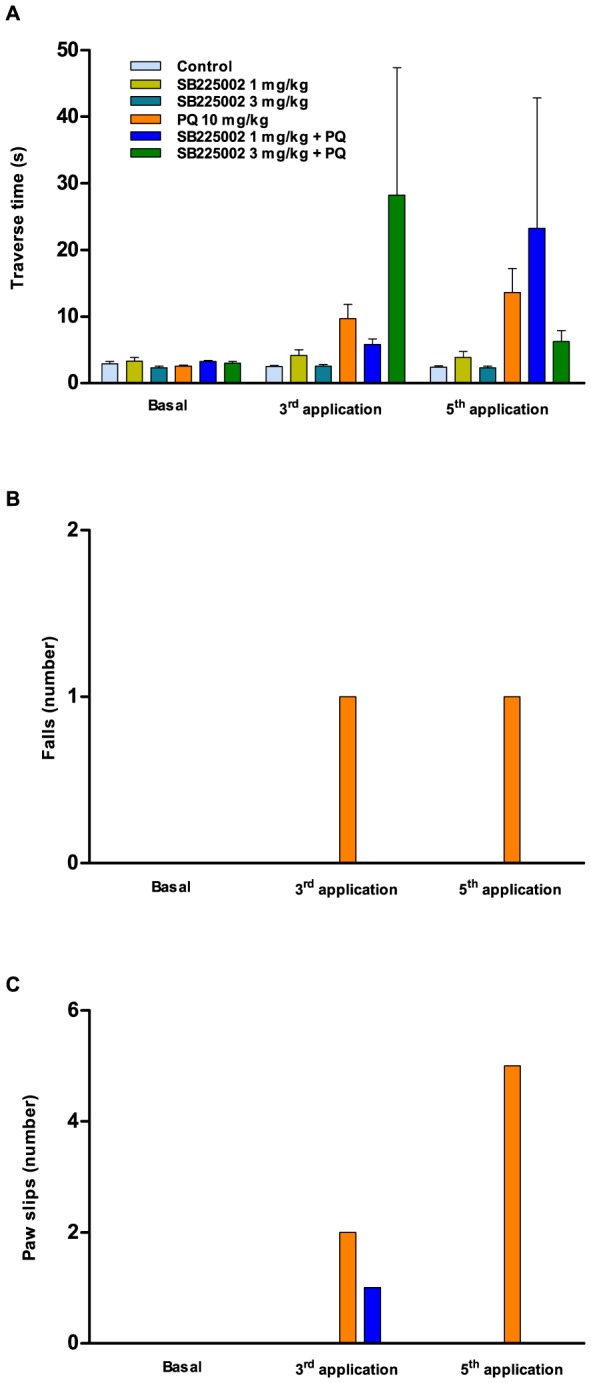
Locomotor skills in the beam-walking test. (A) Time taken to cross the beam in s; (B) number of falls; (C) number of paw slips. Each column represents the mean ± SEM of 4–18 animals per group (ANOVA followed by Bonferroni's post-hoc test was used for analysis of data in Figure 5A).

The motor coordination was firstly evaluated in the beam-walking test. The traverse time was not significantly affected in either experimental group (Figure 5A). During the analysis of falls, one animal (1/18) fell after the 3^rd^ application and another one (1/18) fell after the 5^th^ application (Figure 5B), both belonging to the vehicle/paraquat group. Regarding paw slips, two animals (2/18) from the vehicle/paraquat group presented paw slips after the 3^rd^ application, and five animals (5/18) of this group presented paw slips after the 5^th^ application. One animal from the 1 mg/kg SB225002 plus PQ group (1/9) presented a paw fault at the 3^rd^ application (Figure 5C).

**Figure 6:**
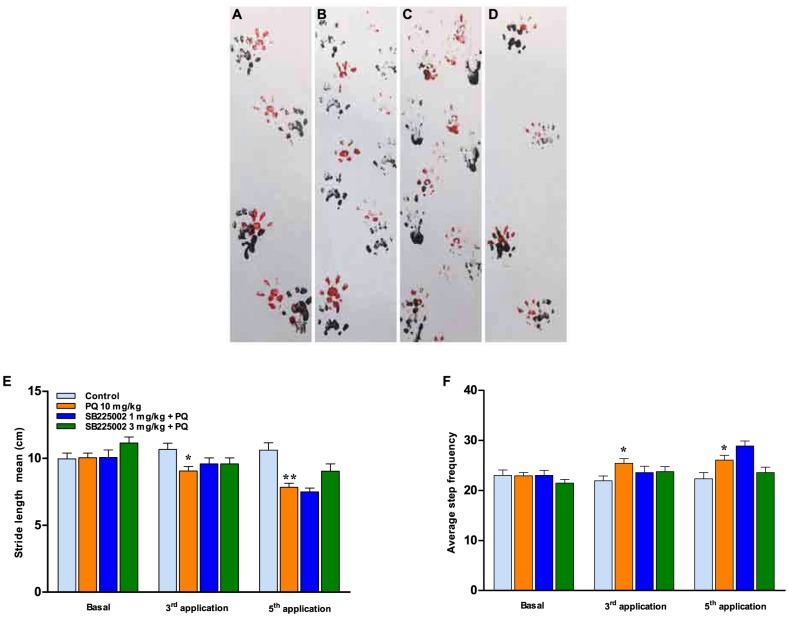
Symptoms of motor dysfunction. Representative walking footprint patterns after the 5^th^ treatment in (A) control (vehicle/saline) group; (B) vehicle/paraquat (PQ)- treated animals; (C) SB225002 (1 mg/kg)/plus PQ group; (D) SB225002 (3 mg/kg)/plus PQ group. The bar graphs depict quantitative analysis of the footprint test regarding the stride length mean (E) and the average step frequency (F). Each column represents mean ± SEM of 9–19 animals per group. ^*^p<0.05 and ^**^p<0.01 compared with control (vehicle/saline) group (ANOVA followed by Bonferroni's post-hoc test).

Next, the animals were evaluated in the footprint model by assessing the stride length mean and the average step frequency. Representative images of footprints after the 5^th^ application are depicted in Figure 6A–D. The administration of paraquat caused a significant reduction of mean stride length (Figure 6E), associated with an increase of step frequency (Figure 6F), after both the 3^rd^ and the 5^th^ applications. Although not significant, effects were observed in the groups pretreated with SB225002 in which the effects of paraquat in motor coordination were virtually prevented by this antagonist, especially at the dose of 3 mg/kg.

### The exposure of paraquat increases inflammatory marker expression in the striatum

**Figure 7:**
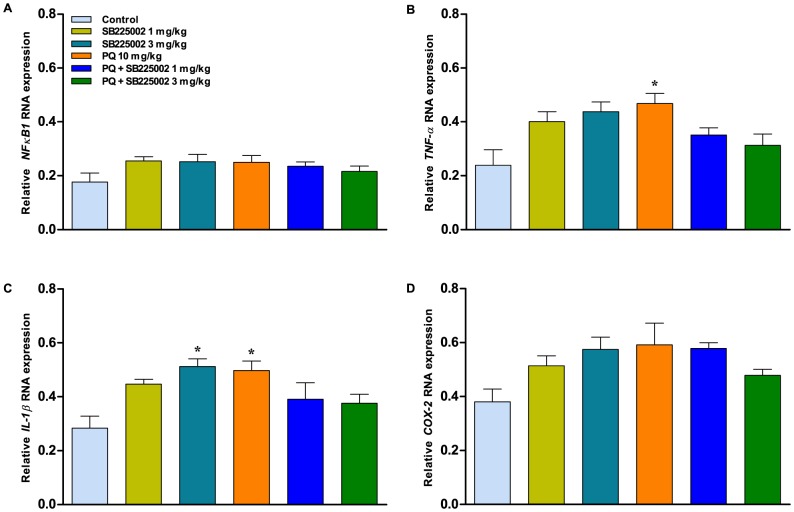
RT-qPCR analysis of inflammatory markers. Relative expression profiles of (A) *NF-κB1*, (B) *TNF-α*, (C) *IL-1β*, and (D) *COX-2*, assessed in the striatum after the 5^th^ paraquat treatment. Each column represents the mean ± SEM, 3–4 samples of striatum. ^*^p<0.05 compared with control (vehicle/saline) group (ANOVA followed by Bonferroni's post-hoc test).

Figure 7 shows the expression profile of some inflammatory markers in the striatum of rats. The relative gene expression of *TNF-α* was significantly increased in the vehicle/paraquat group (p<0.05) in comparison to the control (vehicle/saline) group (Figure 7B). The relative expression of *IL-1β* was increased in the vehicle/SB225002 (3 mg/kg) and vehicle/paraquat (p<0.05) groups when compared to the control (vehicle/saline) group (Figure 7C). Of note, the induction of *TNF-α* and *IL-1β* expression in the striatum was abolished by treatment with SB225002 (Figure 7B and C). *NF-κB1* and *COX-2* mRNA levels did not show any changes in the expression profile amongst different experimental groups (Figure 7A and D, respectively).

### Hematologic changes and neutrophil migration

**Figure 8:**
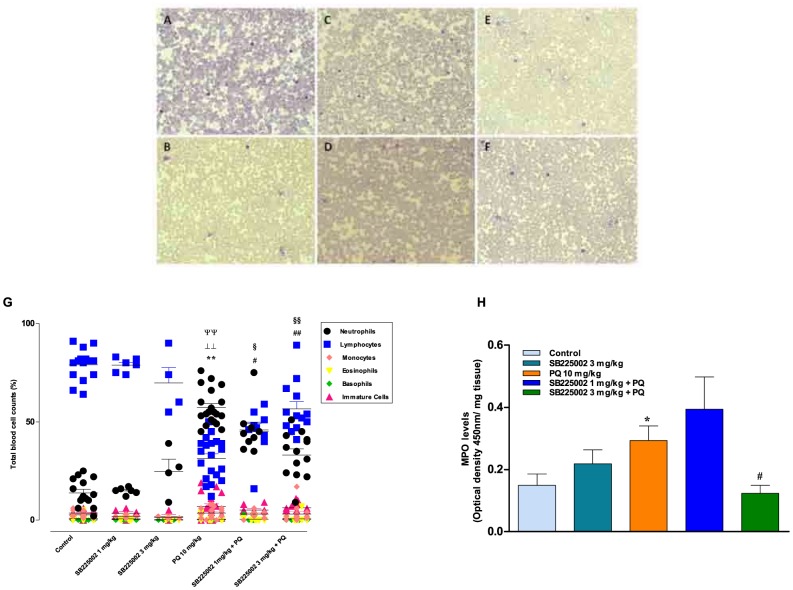
Hematological analysis and lung neutrophil migration. Representative pictures showing the blood smear slides in (A) control (vehicle/saline) groups; (B) vehicle/paraquat (PQ)-treated animals; (C) SB225002 (1 mg/kg)/saline group; (D) SB225002 (3 mg/kg)/saline group; (E) SB225002 (1 mg/kg)/plus PQ group; (F) SB225002 (3 mg/kg)/plus PQ group. Effects of paraquat and SB225002 on the total blood cell counts (G) and neutrophil migration as assessed by MPO activity (H). Each column represents the mean ± SEM of 4–20 animals per group in blood cell counts and 3–12 animals per group in MPO activity. ^**,⊥⊥,ΨΨ^p<0.01 indicate significant differences of neutrophils, lymphocytes, and immature cells in relation to control (vehicle/saline) group, respectively. ^#,§^p<0.05; ^##,§§^p<0.01 indicate significant differences of neutrophils and lymphocytes in relation to vehicle/plus PQ group, respectively. ^*^p<0.05 indicates significant difference of MPO activity in relation to control (vehicle/saline) group; ^#^p<0.05 indicates significant difference to vehicle/plus PQ group (ANOVA followed by Bonferroni's post-hoc test).

At the end of the behavioral experiments, we decided to assess the possible changes of leukocyte populations throughout the different groups. Representative images of different experimental groups are depicted in Figure 8A–F. Total blood cell counts (Figure 8G) revealed a predominance of lymphocytes in the vehicle/saline group and this feature was not significantly modified by SB225002 (1 and 3 mg/kg). Vehicle/paraquat-treated animals showed a predominance of neutrophils, accompanied by a slight increase in the number of immature cells and a reduction of lymphocyte numbers (p<0.01). Pretreatment with SB225002 (1 and 3 mg/kg) was able to significantly modify the effects of paraquat (p<0.05 and p<0.01, respectively), as these experimental groups presented the same percentages of lymphocytes and neutrophils.

Neutrophil migration to the rat lungs was assessed by means of MPO activity. The vehicle/paraquat group presented a significant increase of MPO levels (p<0.05) in comparison to the control group. This parameter was reversed by the co-administration of SB225002 (3 mg/kg) (p<0.05), returning to the control levels (Figure 8H).

## Discussion and Conclusions

The herbicide paraquat is widely employed worldwide, although its use has been prohibited in some countries due to the potential toxic effects [35]. Nevertheless, traces of paraquat can be detected in fruits and vegetables, and even in processed products, which might be related to intoxication events [36]. This agent promotes the formation of ROS and leads to toxicity of the central nervous system (CNS) linked to the loss of dopaminergic neurons, inducing Parkinson-like motor alterations in animal models [17],[37],[38]. The lungs are the major targets of paraquat intoxication, as the herbicide is transported by the polyamine uptake system, accumulating within the alveolar type II epithelial cells, which results in pulmonary fibrosis [39]. In the present study, we evaluated whether the administration of the selective chemokine CXCR2 receptor antagonist SB225002 might prevent either peripheral or central alterations related to paraquat intoxication in rats. We believe that our study contributes to the further understanding of the mechanisms involved in paraquat-induced toxicity, and might open new opportunities to develop potential therapeutic options to treat intoxications caused by this agent.

Initially, we performed a dose-response curve in order to select a sub-lethal scheme of treatment with paraquat using a series of behavioral evaluations. Accordingly, Czerniczyniec et al. [18] demonstrated that administration of paraquat (10 mg/kg, i.p.) in rats, dosed weekly for a month (40 mg/kg overall), resulted in behavioral changes associated with brain mitochondrial dysfunction, which corroborates our choice of protocol.

The schedule of treatment with paraquat adopted by us led to a time-related reduction of body weight and rectal temperature in rats, which is in accordance with previous literature [40],[41],[42],[43],[44]. Although the treatment with SB225002 (3 mg/kg, i.p.) was able to produce only a partial effect on body weight loss, the same dose of this antagonist largely prevented the hypothermia induced by paraquat. This series of results indicates that pharmacological inhibition of CXCR2 receptors by SB225002 can provide protective effects against some complications related to paraquat poisoning. Of high interest, the study conducted by Bento et al. [13] demonstrated that repeated administration of SB225002, at doses as low as 0.3 mg/kg, was effective in markedly preventing the body weight loss in the mouse model of TNBS-induced colitis. Nonetheless, to our knowledge, there is no previous report showing the ability of SB225002 to modulate hypothermia, thus we reveal a novel effect for this antagonist. In spite of that, chemokines and their receptors have been detected in most cell types in CNS [45],[46],[47] (for review see: [48]), which might help to explain our data.

Among the symptoms of paraquat poisoning, it is relevant to cite the occurrence of intense abdominal pain in clinics [49],[50]. The administration of paraquat resulted in a marked and time-related increase of nociception scores, and pretreatment with SB225002 significantly reduced paraquat-elicited nociception at all evaluated timepoints, displaying an apparent dose-related profile. It is well known that paraquat toxicity is likely related to oxidative stress [51], and generation of ROS by this herbicide is able to modulate neutrophil function [52]. Remarkably, prior experimental evidence showed that visceral pain elicited by colorectal distention relies on oxidative stress [21]. Thus, we hypothesize that inhibition of neutrophil migration by SB225002 might prevent the complications originating from paraquat-induced ROS generation, including the abdominal discomfort. Corroborating our data, it was previously demonstrated that treatment with SB225002, in doses ranging from 0.3 to 3 mg/kg, significantly inhibited the visceral nociception induced by acetic acid in mice [14] or by cyclophosphamide in rats [15].

We also aimed to investigate whether the treatment with SB225002 would prevent motor deficits induced by paraquat intoxication in rats. In fact, it has been largely demonstrated that paraquat exposure is associated with serious motor impairments that originate from oxidative stress and neurotransmission deficits in several brain regions (for review see: [37],[53]). In this study, the exposure to paraquat led to decreased locomotor and exploratory activities (open-field test), which is in agreement with previous literature [38],[44],[54],[55]. Furthermore, a reduction of fecal bolus number was also observed in paraquat-treated rats, as demonstrated before [36]. Furthermore, the administration of paraquat resulted in worsened performance in beam-walking and footprint paradigms. A series of alterations related to locomotor dysfunction were observed in previous publications dealing with the consequences of paraquat poisoning, mainly via mechanisms involving dopaminergic neurotoxicity [17],[35],[44],[49],[51],[56]. Concerning the treatment with SB225002, this antagonist presented only partial effects on paraquat-induced motor deficits, although a general improvement of coordination skills was achieved, according to assessment in the beam-walking test or the footprint model. As mentioned beforehand, chemokine receptors are expressed throughout different brain regions [48], which might support the beneficial effects of SB225002 in our experimental paradigm, although the ability of this antagonist to cross the blood-brain barrier remains to be further investigated. Furthermore, it is possible to surmise that prevention of peripheral inflammation by SB225002 might avoid paraquat-induced oxidative stress within the CNS, contributing to the positive effects of this antagonist on motor performance.

It has been previously shown that the striatum is considered the most important site of dopamine action and motor control [57]. For this reason, the striatum was used in the transcriptional analysis of known molecular markers of inflammation. The expression profile of nuclear factor of kappa light polypeptide gene enhancer in B-cells 1 (*NF-κB1*), tumor necrosis factor-α (*TNF-α*), interleukin-1β (*IL-1β*), and cyclooxygenase-2 (*COX-2*) was determined. *TNF-α* and *IL-1β* expression was induced in the striatum after paraquat administration, which, in turn, was abolished by treatment with SB225002. It is important to highlight that *TNF-α* expression is persistent in inflammatory response [29] and *IL-1β* is responsible for recruitment and activation of other immune cells at the inflammatory site (for review see: [58]).

Considering the evidence discussed above, we decided to examine to what extent the administration of SB225002 might modulate peripheral inflammation induced by paraquat exposure. Strikingly, the repeated application of paraquat, resulted in a marked reduction of blood lymphocytes in comparison to saline-treated control animals, accompanied by an increase of neutrophil counts, and the scarce presence of immature cells. More interestingly, SB225002-treated groups (with either 1 or 3 mg/kg) presented the same percentages of lymphocytes and neutrophils, indicating the ability of the tested antagonist to reverse the peripheral inflammation elicited by paraquat. Similar to our data, Aires et al. [59] recently demonstrated that an acute single administration of paraquat (20 mg/kg, i.p.) resulted in marked neutrophilia, an effect that was prevented by the TNF-α inhibitor etanercept. Aligned to these experimental pieces of evidence, an attractive clinical study recently published by Fareed et al. [60] highlighted the gravity of hematological alterations observed in agricultural workers exposed to some pesticides. Furthermore, a recent publication by Kang et al. [61] demonstrated a correlation between the absolute lymphocyte counts and the 30-day mortality rates after paraquat poisoning in humans, suggesting an immunomodulatory action for this herbicide. Whether or not the therapy with anti-inflammatory agents (such as SB225002) might prevent the clinical complications related to paraquat intoxication needs additional investigation.

Lungs are well recognized as the main targets of paraquat intoxication [62]. Paraquat-induced lung toxicity is likely dependent on development of pulmonary edema, inflammatory cell infiltration, and damage of alveolar cells, resulting in fibrosis [63]. A study conducted by Venkatesan [64] demonstrated that single administration of paraquat at a high dose (50 mg/kg, i.p.) led to a significant increase of lung neutrophil migration in rats, as indirectly measured by means of MPO activity. Furthermore, the acute exposure to paraquat (20 mg/kg, i.p.) produced a marked increase of neutrophil influx to the broncoalveolar lavage of mice, and this parameter was reduced by TNF-α inhibition [59]. In the present study, the repeated i.p. administration of paraquat caused a significant increase of lung MPO activity, which was prevented by pretreating animals with SB225002 at 3 mg/kg.

In summary, on the basis of results present herein, it is possible to suggest that pharmacological inhibition of CXCR2 receptors by the antagonist SB225002 is able to interfere, in a dose-dependent manner, with paraquat-related systemic toxicity. Further studies are necessary to reinforce our findings.
